# Electrical and Optical Properties of Co_75_Si_15_B_10_ Metallic Glass Nanometric Thin Films

**DOI:** 10.3390/ma14010162

**Published:** 2020-12-31

**Authors:** Danagul Shauyenova, Sol Jung, Haneul Yang, Haein Yim, Heongkyu Ju

**Affiliations:** 1 Department of Physics, Gachon University, Seongnam, Gyeonggi-do 13120, Korea; dana.shauyenova@gmail.com; 2 Department of Physics, Sookmyung Women’s University, Seoul 04310, Korea; 73461928@naver.com (S.J.); didgksmfzzz@gmail.com (H.Y.)

**Keywords:** thin films, amorphous alloys, Co-based metallic glasses, electrical and optical characterization, microscaled devices

## Abstract

Co-based (Co_75_Si_15_B_10_) thin-film metallic glass (TFMG) with nanometric thicknesses (100~300 nm) was investigated for its structural, electrical, and optical properties. The TFMG structure was examined using scanning electron microscopy and X-ray diffraction, while electrical properties were examined using inductance/capacitance/resistance spectroscopy, cyclic voltammetry, and Hall effect measurements. In addition, optical absorption/reflection/transmittance measurements were performed to examine optical properties. Results revealed that Co-based TFMGs, which have an amorphous structure without surface defects, behave like a dielectric material, with higher resistivity and much lower carrier concentration than pure cobalt (Co) thin films of the same thickness, despite its mobility being modestly larger than its Co counterparts. Meanwhile, the optical investigation of TFMG enabled us to determine the complex relative permittivity (complex relative dielectric constant) $\tilde{\epsilon_{r}}$ at a visible wavelength (632.8 nm). Moreover, unlike normal metals, TFMGs exhibited a large positive value of the real part of $\tilde{\epsilon_{r}}$, while exhibiting properties of substantial absorption of light (absorption coefficient α). It was also found that the Co-based TFMG gained optical transparency for thicknesses less than 5 nm. TFMGs demonstrated the nearly thickness-independent properties of the electrical and optical parameters probed, a feature of high-index, dielectric-like material with negligible size effects, which may have applications in micrometer-scaled optoelectronic and magneto-optical devices.

## 1. Introduction

Metallic glass (MG), a solid medium with an amorphous structure of disordered metal atoms, has attracted continuous interest due to several advantages largely stemming from its structural homogeneity [1]. Since the first discovery of the MG that contained 75 at.% Au and 25 at.% Si by a group at Caltech [2], various MGs have been studied. The inherent characteristics of MG include its negligible size-effects of electromechanical properties [3], high thermal stability [4], high strength at low temperatures, as well as high flexibility at high temperatures [5], large elastic limit [6], and excellent corrosion resistance [7,8]. Use of bulk metallic glass (BMG), its formation optimized by engineering a cooling rate for melt quenching, has lent itself to the aforementioned benefits, thus making it amenable to commercialization [9,10].

The structural homogeneity of MG, which would allow minimal size-effects, prompted its new class with reduced dimensions to be studied (i.e., a thin-film metallic glass (TFMG) with thickness of hundreds of nanometers) for various applications in micro- and nano-device technologies [11,12,13,14]. Accordingly, the relevant fabrication methods advanced from liquid-to-solid (in cases of BMG) to vapor-to-solid deposition such as via evaporation and sputtering [15,16]. The combination of different atoms has been used for TFMGs to engineer both optoelectronic and mechanical properties. Binary Zr-Cu based TFMGs have been reported to exhibit conductivity 1.4 and 8 times as large as that for the pure Zr and Cu films, respectively [17]. Meanwhile, it was demonstrated that Pd-based TFMGs were optically transparent at thickness less than 10 nm while being mechanically flexible [18]. Furthermore, Zr-Cu-Al-based TFMG was also reported to produce enhanced transmittance at near-infrared wavelengths by the addition of Si [19]. 

Cobalt (Co)-rich amorphous alloys fall into the ferromagnetic class, similar to Fe and Ni composites. They exhibit magneto-elastic and soft magnetic properties [20,21,22], making them attractive as possible next-generation magnetic recording media. Co-based TFMGs have been reported to have high magnetic induction and high permeability at MHz frequencies [23]. In particular, anisotropic magnetization available with Co-based TFMGs (75 percent Co), such as monolayer CoSiB and multilayered CoSiB/Pt and CoSiB/Pd, has been investigated [24,25,26]. Potential use of this magnetic TFMG would prompt its integration into optoelectronic components for magneto-optic or magneto-electronic devices, thus requiring basic knowledge of its optoelectronic properties. However, few reports have described the electrical and optical properties of Co-based TFMGs for such magneto-optoelectronic applications.

In this study, we examined the electrical and optical properties of CoSiB TFMGs with thicknesses ranging from 5 to 300 nm sputtered on a glass substrate with a surface area of 1.4 cm^2^. It was found that, unlike normal metals, TFMG exhibited a very large magnitude and positive value of the real part of the relative permittivity (relative dielectric constant) $\tilde{\epsilon_{r}}$ while having the properties of substantial absorption of light at the visible wavelength. The electrical and optical properties of TFMG demonstrate its nearly independent thickness, a feature suited for applications where little size effects are required, such as in micrometer scaled optoelectronic devices/magneto optical devices.

## 2. Materials and Methods 

### 2.1. Deposition of CoSiB TFMG and Co Thin Film

A CoSiB (75:15:10 at atomic percent) thin film metallic glass was sputtered on a glass substrate using a 3-inch, 6-gun-type DC magnetron sputtering system (YISP-0206T, Yein-Hitech). This system consists of a load-lock chamber that mounts a sample and inserts it into the main chamber using a magnetic bar, and a main chamber in which sputtering is performed. The wafer was rotated in a predetermined direction by an internal motor, and the specimen was positioned directly on the target to be deposited with an optical sensor using a rotating substrate coaxially connected to the substrate holder. During the experiment, the distance between the target and the substrate was kept at 8 cm. The sputtering process was conducted at room temperature using an argon pressure of 0.27 Pascal and 2.3 Å/s deposition rate conditions to obtain all TFMGs with thicknesses ranging from 5 to 300 nm, using a CoSiB single target purchased from Assemtech Co., Ltd. In this case, a soda lime glass with an area of 1.4 cm × 1.4 cm was used as a transparent substrate for all samples probed. Subsequently, to remove organic or foreign substances on the surface, they were ultrasonically washed for 10 min each in the order of acetone, ethyl alcohol, and tertiary distilled water, and then dried.

For the control experiment, pure Co thin films were coated on a glass substrate at room temperature using a radio-frequency sputtering system (Daeki hi-tech Co., Ltd., Daejeon, Korea). The substrate used as the sample was prepared by cutting soda lime glass into a size of 1.4 cm × 1.4 cm. The cleaning process of substrate sample was same as for the TFMG samples. During thin-film deposition, the vacuum sputtering process was maintained at 0.39 Pascal using a 99.9% Co target purchased from iTASCO. In both spattering cases, Ar gas was used to maintain the vacuum level and to form plasma. The deposition rate was about 2.87 Å/s.

### 2.2. Characterization Methods

We checked the structural properties of the CoSiB TFMG and pure Co thin film using an X-ray diffraction device (D8 Advance Trio/Twin, Bruker, Karlsruhe, Germany) operated in a grazing incidence geometry. The range of the diffraction angle 2θ was set from 10 to 110 degrees. For surface morphological characterization, we took images of the surface using a field-emission scanning electron microscope (SEM) (FE-SEM, JEOL JSM-7500F). 

**Figure 1:**
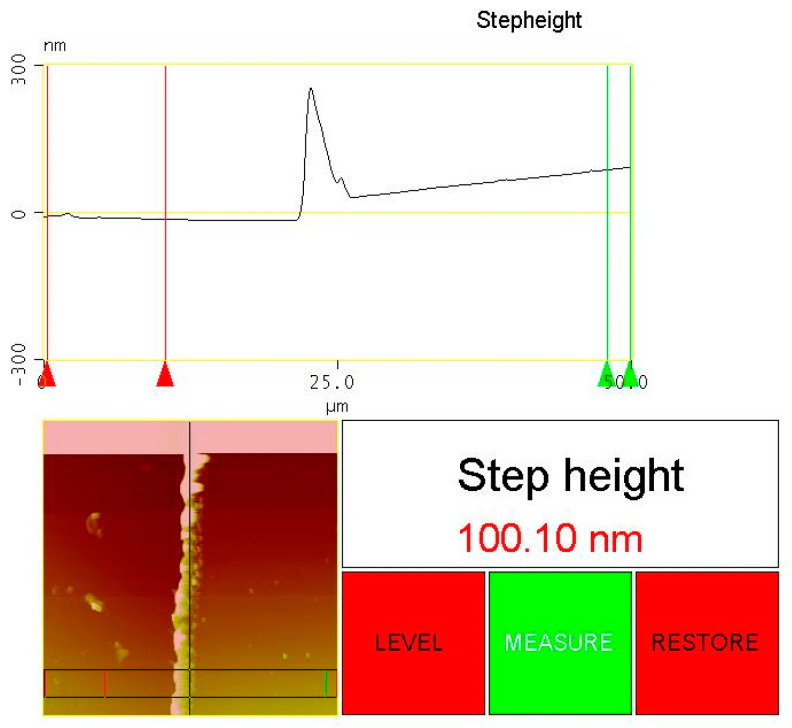
Thickness measurements of CoSiB thin film (100 nm) on glass substrate using AFM.

The film morphology and thickness information were obtained using an atomic force microscope (AFM) (Model No. 920-006-101, Veeco Metrology System, North Bergen, NY, USA). To determine the thickness of the TFMG, we arranged several samples, each with its surface half sputtered and the other half unsputtered. The film thickness was estimated by measuring the difference in surface height between the thin-film coated part (red lines) and the bare glass part (green lines) on a substrate, as shown in Figure 1.

We measured the electrical properties of the TFMG with three different thicknesses (100, 200, 300 nm; i.e., the frequency-dependent inductance/capacitance/resistance/impedance) using a four-point probe LCR meter (PSM 1735, Newton4th Ltd., Loughborough, UK). Then, they were compared with those of pure Co thin films at the corresponding thicknesses. Electrical charge transport-relevant parameters, such as carrier concentration, mobility, resistivity, and Hall coefficients, were obtained from the Hall effect measurements (HSM-3000, Ecopia). 

The electrochemical measurements were performed using a Potentiostat equipped with a conventional three-electrode system (VersaSTAT, AMETEK, Berwyn, PA, USA). The given TFMG on a glass substrate was used as a working electrode (electroactive surface area of 1.4 cm^2^), while the Ag/AgCl and platinum electrodes were used as the reference and counter electrodes, respectively. The electrolyte was a 1 M potassium hydroxide (KOH) solution. 

**Figure 2:**
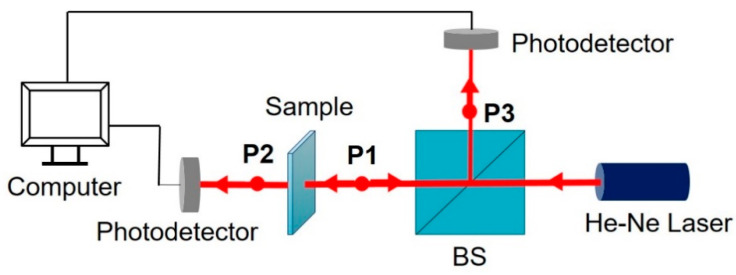
Schematic of the setup for measuring optical transmittance and reflectance for the thin-film metallic glass (TFMG).

For optical characterization of the TFMG, we first measured transmittance spectra of light through the TFMG using a UV-VIS spectrophotometer (REGOL, Ultra-3000) at the visible-near infrared wavelengths of 400–800 nm. In addition, for the purpose of obtaining the $\tilde{\epsilon_{r}}$ of the TFMGs, we built an optical setup to measure reflectance and transmittance, as shown in Figure 2. The light source was an He-Ne laser at a wavelength of 632.8 nm (25-LHP-991-230, Melles Griot). A 50:50 optical beam splitter (BS) (CCM1-BS013, Thorlabs) split a laser into two beams of equal optical powers, with only part of the transmitted beam being incident on the TFMG sample. The light reflected from the sample would be collected by the reflection port of the 50:50 ratio BS. As shown in Figure 2, we measured the optical power incident on the sample, i.e., $P_{in}$ at the point P1, while transmitted optical power $P_{out}$ could be measured at P2. The optical power reflected from the TFMG sample (i.e., $P_{\mathrm{ref}}$) could be estimated by doubling the power measured at P3. Measurement of optical powers at the three different positions enabled us to estimate the real and imaginary parts of the ${\tilde{\epsilon }}_{r}$ of the TFMG with different thicknesses, as discussed later.

## 3. Results and Discussion

### 3.1. Structure Characterization

**Figure 3:**
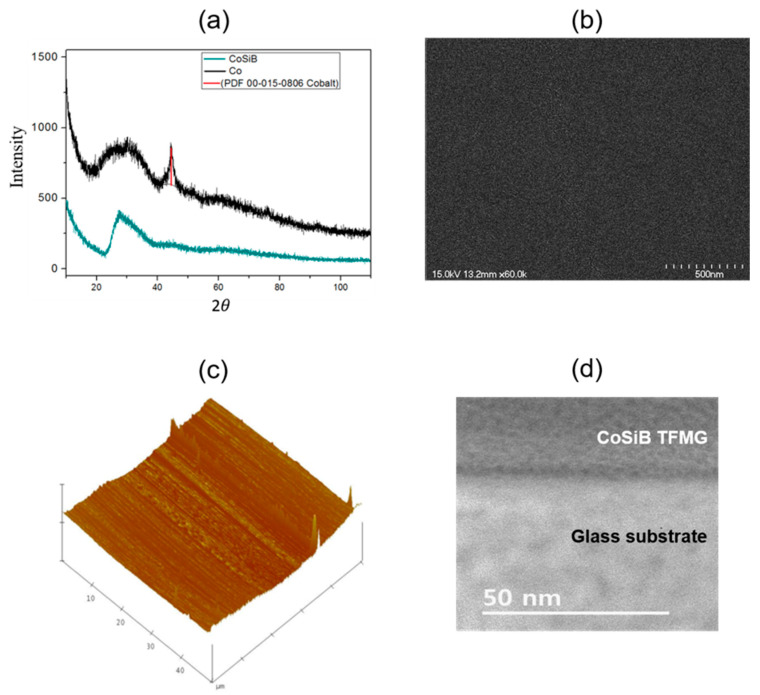
Structural and morphology characterization CoSiB TFMG: (**a**) the X-ray pattern of the CoSiB TFMG and Co film (100 nm thick TFMG). (**b**) An SEM image of the surface of the CoSiB TFMG captured using a field-emission SEM (100 nm-thick TFMG). (**c**) Morphology scanning and thickness measurements by AFM (100 nm thick TFMG). (**d**) A TEM image of the cross-section across the film–substrate boundary of the 200 nm thick TFMG.

Figure 3a shows the X-ray diffraction patterns for the TFMG of 100 nm thickness coated on a glass substrate. The absence of any distinct peak, except for the broadened one induced by the glass substrate at around 25–35 degrees, indicated its fully amorphous structure. These features were also found in the samples of the other TFMG thicknesses (200 nm, 300 nm). We also measured X-ray diffraction of a pure Co film of 100 nm thickness, revealing the fcc (face-centered cubic) structure confirmed by the peak between 40 and 50 degrees, with the glass substrate induced broadened peak, as shown in Figure 3a. Figure 3b shows an SEM image of the surface of the 100 nm thick TFMG captured using a field-emission SEM. This image exhibited neither visible pores nor distinguishable cracks in the structure, indicating the rather smooth surface of the amorphous structure with good continuity. Figure 3c shows the AFM image of the TFMG sample (100 nm thickness). The scan size was set to 50 μm (*x* axes 10 nm/dev, *z* axes 70 nm/dev), and the scan rate was 1 Hz. Its surface morphology enabled us to estimate a surface roughness of ~6.4 nm, implying a reasonably smooth surface. Figure 3d shows a TEM image of the cross-section across the film-substrate boundary for a TFMG 200 nm thick. It supports the presence of an amorphous structure with neither visible pores nor distinguishable cracks.

### 3.2. Characterization Using an LCR (Inductance, Capacitance, and Resistance) Meter

**Figure 4:**
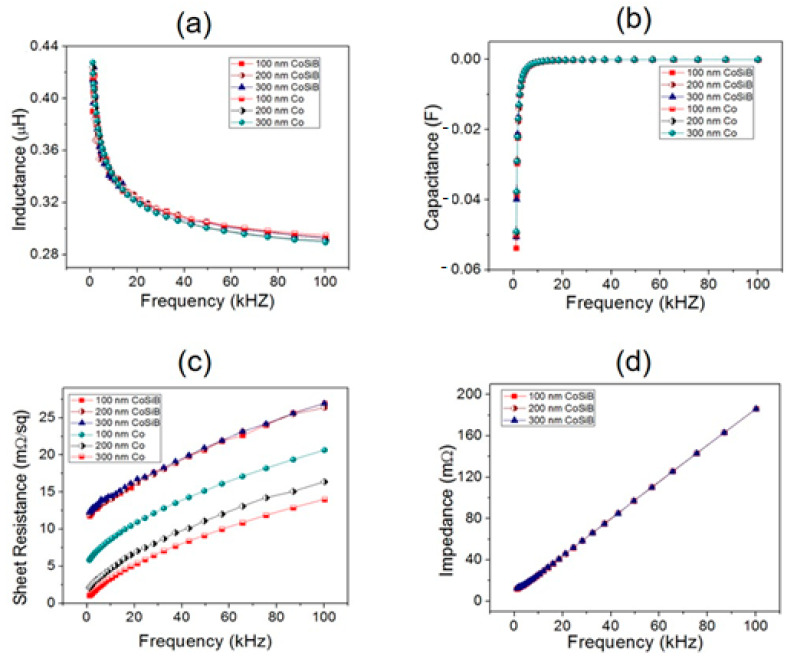
Four-probe electrical impedance component measurements in frequency ranges from 100 Hz to 100 kHz: (**a**) inductance, (**b**) capacitance, and (**c**) sheet resistance spectroscopies for the TFMG samples and Co thin film of different thicknesses. (**d**) Impedance spectroscopy of CoSiB TFMG of three different thickness.

We measured the inductance, capacitance, resistance, and impedance of TFMGs of three different thicknesses (100, 200, and 300 nm) as a function of operation frequency using an LCR meter/impedance analyzer (PSM17100, PsimetriQ, Newton 4th) via a four-wire Kelvin connection. The frequency range was set from 100 Hz to 100 kHz. For comparison, we also measured properties of the pure Co thin films of the corresponding thicknesses. No significant difference in inductance/capacitance was observed between the CoSiB TFMG and pure Co film for all the thicknesses, as shown in Figure 4a,b. This indicated that the TFMG could still possess magnetic properties similar to the pure Co thin film, suggesting the possible use of CoSiB TFMG for an inductive component.

Figure 4c shows the frequency-dependent sheet resistance of the CoSiB TFMG and the pure Co thin film for the three thicknesses (100, 200, 300 nm). As expected, the sheet resistance of the pure Co thin film decreased with increasing thickness. It was observed that, for each thickness, the sheet resistance of the CoSiB TFMG exceeded that of the pure Co thin film at frequencies between 0.1 kHz and 100 kHz. At the lowest frequency probed (0.1 kHz), the 300 nm thick TFMG exhibited 10-fold greater (>10 mΩ/sq) resistance than the Co thin film (~1 mΩ). Similar behavior was also found for the other two thicknesses (100 and 200 nm) with the enhancement of more than a couple of fold. However, it was interesting to note that the sheet resistance of the CoSiB TFMG film did not vary significantly with thickness, unlike the pure Co thin film. This minimal dependence of electrical properties on thickness could evolve to negligible size effects, largely due to the structural homogeneity of TFMGs. It was also found that alloying Co with Si and B of 10–20 atomic % into an amorphous nanostructure increased the sheet resistance compared to the pure Co thin film of the same thickness.

Figure 4d shows the frequency-dependent impedance of the CoSiB TFMG for the three different thicknesses; its behavior as a function of frequency was similar to the frequency-dependent resistance (Figure 4c). This was due to dominant effects of the resistance over the capacitive and inductive reactance in impedance magnitude. Minimal dependence of the aforementioned electrical properties on thickness would make it possible to use the CoSiB TFMG as material for micrometer-scaled electronic devices, which requires no size-dependent effects [3]

### 3.3. Hall Measurements at Room Temperature

Hall measurements were performed at room temperature with the CoSiB TFMG and pure Co thin films for the three different thicknesses, as shown in Table 1. It is noteworthy that the TFMG exhibited a couple of times higher mobility while having more than ~10 times lower carrier concentration, leading to several times higher resistivity and ~10 times higher Hall coefficient than the crystalline counterpart (Co-thin film) for all thicknesses probed. The lower conductivity of TFMG could be attributed to the much lower carrier concentration despite the mobility being modestly higher than the Co thin film. It was also noted that the thickness dependence of mobility in the TFMG was apparently relatively weaker (all above 240 ${\mathrm{cm}}^{2}/\mathrm{Vs}$) than that of the crystalline Co thin film. The higher Hall coefficients of TFMG were believed to be due to anomalous Hall effects that also dominated the Hall effects occurring in the ferromagnetic materials similarly to the Co thin films [20,21,22]. These properties enabled us to propose a potential use for TFMG as ferromagnetic dielectric-like material.

### 3.4. Electrochemical Measurements

**Figure 5:**
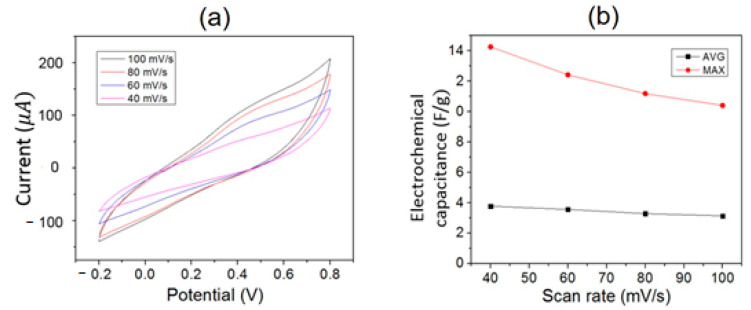
Electrochemical measurements for 100 nm CoSiB TFMG: (**a**) electrical current versus potential at different scan rates. (**b**) Average (AVG) and maximum (MAX) values of capacitance versus voltage scan rate, extracted from (**a**).

Cyclic voltammetry (CV) was used to probe chemical reactions via flows of electrode electrons controlled by a potentiostat that provided the scanned external voltage. We employed CV to examine the electrochemical properties of CoSiB TFMG with a 1M KOH electrolyte solution at room temperature. The voltage ranged from—0.2 V to 0.8 V, while the potential scan rate was set from 40 mV/s to 100 mV/s. Figure 5a presents the electrical current versus potential obtained by the CV measurement with the TFMG as a working electrode. Its electrochemical response included oxidation or reduction when the potential was swept positively or negatively. Higher scan rates produced larger current. However, the voltammograms obtained experimentally for various scan rates contained a current peak neither at the anodic nor at the cathodic trace, in contrast to the electrochemical properties of Co [27]. They featured a behavior similar to the capacitance-like electrode nature, reflecting the dielectric-like properties of the TFMG as already observed by the resistivity given by the Hall effects results mentioned above. Capacitance, which is proportional to the current divided by the scan rate, could be extracted from the voltammograms as shown in Figure 5b. The TFMG electrochemical capacitance per unit mass (farad per gram, F/g) decreased with the scan rate, with a maximum of 14 F/g at 40 mV/s.

### 3.5. Optical Properties

**Figure 6:**
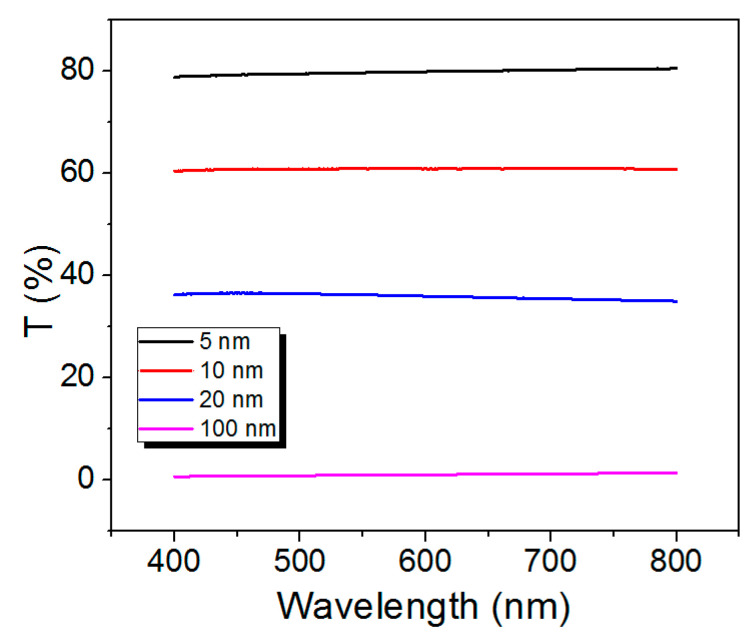
Optical transmittance (T) of a CoSiB TFMG of the different thickness (5, 10, 20, 100 nm) at wavelengths scanned from 400 nm to 800 nm using a UV-VIS spectrophotometer.

Figure 6 shows the optical transmittance spectra of light through the CoSiB TFMG of 5, 10, 20, and 100 nm thicknesses, measured using a UV-VIS spectrophotometer in which the probe wavelength range was set from 400 nm to 800 nm. The 100 nm thick TFMG exhibited almost zero transmittance over all wavelengths probed without any spectral peak of absorption of light. This prompted us to check the transmittance spectra of light through the thinner TFMGs (i.e., those of thicknesses of 5, 10, and 20 nm). It was found that the TFMG gained optical transparency for thicknesses less than 5 nm, unlike the Pd-based TFMG [18]. 

The transmittance spectra exhibited no spectral peak of absorption, but uniform attenuation over all wavelengths, as shown in Figure 6. The optical dispersion principle in the Kramer–Kronig relation dictated the absence of a spectral peak of absorption leading to the presence of the refractive index n, which would be nearly constant over the wavelength range probed (400–800 nm). 

In order to find the complex value of ${\tilde{\epsilon }}_{r}$ of TFMGs of 100, 200, and 300 nm thickness at a visible wavelength, we investigated the optical transmission, absorption, and reflection at a normal incidence to its surface at the He-Ne laser wavelength (632.8 nm) using the optical setup mentioned above (see Figure 2).

**Figure 7:**
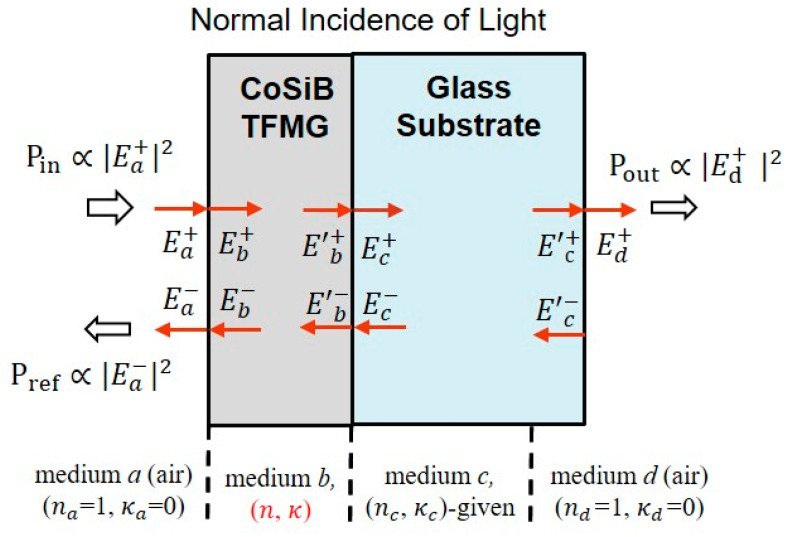
Schematic of light propagation through the TFMG (deposited on a glass substrate) at normal incidence. *n* and *κ* represent the refractive index and extinction coefficient, respectively.

We used the transfer matrix approach [28,29] to estimate the $\tilde{\epsilon_{r}}$ of TFMG deposited on a glass substrate assuming normal incident of light, as shown in Figure 7. The $E_{i}^{+}$, $E_{i}^{-}$, $E_{i}^{\prime +}$, and $E_{i}^{\prime -}$ (i=a,b,c,d) represent the electric fields of light in each layer, i.e., air(*a*), TFMG (*b*), a glass substrate (c), and air (*d*), near the interfaces (see Figure 7). The primed E denotes the field that gains the optical phase developed during the forward propagation through the layer, while the unprimed E denotes the field that gains the optical phase during backward propagation through the layer. The plus sign denotes the propagation direction from left to right, while the minus sign denotes from right to left. Boundary conditions for electromagnetic fields (e.g., the continuity of surface-parallel components of electric and magnetic fields across each interface) enabled both the electric field transmitted into air on the right and reflected back into air on the left (i.e., $E_{d}^{+}$*,* $E_{a}^{-}$) to be calculated. Thus, $T=P_{out}/P_{in}={\left|t\right|}^{2}$ and $R=P_{ref}/P_{in}={\left|r\right|}^{2}$ give the optical transmittance and reflectance, where $t=E_{d}^{+}$/$E_{a}^{+}$ and $r=E_{a}^{-}$/$E_{a}^{+}$ are the transmission and reflection coefficients, respectively. Optical absorption A, defined as the ratio of optical power absorbed by the sample relative to $P_{\mathrm{in}}$, could be estimated by measuring both T and R with energy conservation of 1=A+T+R. Given the fact that R and A were functions of the real and imaginary parts of $\tilde{\epsilon_{r}}$ of all the layers that light propagated through, the values experimentally obtained for R and A could lead us to estimate the complex value of $\tilde{\epsilon_{r}}$ of TFMG (assuming no imaginary part of glass $\tilde{\epsilon_{r}}$). Here, we ignore the optical power loss induced by light scattering due to the surface imperfection such as roughness and defects. Thus, this gave the relation $A=e^{-\alpha z}$. Here α is the absorption coefficient associated with the extinction coefficient $\kappa =\alpha /\left(2k_{0}\right)$, where $k_{0}\;$ is the wavenumber of light in vacuum. 

Table 2 reports the optical properties R, T, and A and optical characteristic parameters of the TFMGs. The large positive value of the real part of $\tilde{\epsilon_{r}}$ indicates that the TFMG could be, unlike normal metals, considered a dielectric-like material, as in agreement with the electrical properties mentioned above. In addition, the large positive value of the imaginary part of $\tilde{\epsilon_{r}}$ of the TFMG implies substantial absorption of light at 632.8 nm, being in agreement with Figure 6, which shows the non-transparent film of thicknesses greater than 100 nm at visible wavelengths. It is worth noting that the values listed in Table 2 would be approximately valid at other visible wavelengths. This is due to the index dispersion relation governed by the Kramer–Kronig relation, which indicates little change in refractive index over spectral regions where no resonant absorption of light occurs, as shown in Figure 6 [30].

## 4. Conclusions

We investigated the structural, electrical, and optical properties of Co_75_Si_15_B_10_ TFMG of various thicknesses (100, 200, 300 nm) deposited on a glass substrate. The XRD measurements revealed its fully amorphous structure, while its SEM and AFM images confirmed a rather smooth surface with good continuity. The methodology of electrical characterization of TFMGs includes inductance/capacitance/resistance spectroscopy, cyclic voltammetry, and Hall effect measurements, showing the higher resistivity due to lower carrier concentration than pure cobalt (Co) thin films of the same thickness. The TFMGs reflect the thickness independence of such electrical properties, and the ferromagnetic properties as checked by the inductance spectroscopy and anomalous Hall effects. This may open up applications in which Co_75_Si_15_B_10_ TFMG may find use in micro-scaled electronic/magneto-electronic devices. 

We also investigated TFMGs using optical absorption/transmission/reflection measurements to obtain the complex values of $\tilde{\epsilon_{r}}$ at 632.8 nm. This complex value can also be approximately valid at other visible wavelengths, given the fact that there is no spectral peak of absorption of light over the visible wavelength range (thus little spectral dependence of refractive index). It was revealed that the TFMGs have large, positive values of the real part of $\tilde{\epsilon_{r}}$ with its imaginary part quite large, unlike normal metal. This permits the TFMG to fall into a quasi-dielectric material that is in non-transparent to visible wavelengths. The spectral transmission of light through the TFMGs also indicates that the TFMG films can become transparent at thicknesses ≤5 nm. These optical properties can be used as relevant information when TFMGs are integrated into optoelectronic devices. 

Future work may include a comparative study of electrical and optical properties of TFMGs and Co-based thin films, all fabricated using temperature-controlled protocols.

## Figures and Tables

**Table 1:** The results of room temperature Hall measurements with the CoSiB TFMG and pure Co thin film.

Thickness [nm]	Sample	Carrier Concentration [/$cm^{3}$]	Mobility [$cm^{2}$/Vs]	Resistivity [Ωcm ]	Hall Coefficient [$m^{2}$/C]
100	CoSiB	1.107 $\times 10^{20}$	241.3	$2.337\times 10^{-4}$	$5.639\times 10^{-2}$
	Co	2.145 $\times 10^{21}$	73.30	$3.969\times 10^{-5}$	$2.910\times 10^{-3}$
200	CoSiB	$1.090\times 10^{20}$	242.9	$2.356\times 10^{-4}$	$5.724\times 10^{-2}$
	Co	$0.900\times 10^{21}$	119.2	$5.814\times 10^{-5}$	$6.932\times 10^{-3}$
300	CoSiB	$0.890\times 10^{20}$	252.2	$2.780\times 10^{-4}$	$7.012\times 10^{-2}$
	Co	$1.028\times 10^{21}$	87.0	$6.980\times 10^{-5}$	$6.072\times 10^{-3}$

**Table 2:** Estimated optical properties of CoSiB amorphous thin films at 632.8 nm.

	Thickness (nm)	R	T	A	α	$\tilde{\epsilon_{r}}$
CoSiB	100	0.4651	0.0001	0.5348	$2.98\times 10^{7}$	13.94 +1.12i
	200	0.4496	0.0011	0.5493	$6.16\times 10^{6}$	16.40 + 2.52i
	300	0.4474	0.0111	0.5415	$1.99\times 10^{7}$	15.23 + 0.15i

## Data Availability

The data presented in this study are available on request from the corresponding author. The data are not publicly available due to privacy.
